# Mature T-Cell leukemias: Challenges in Diagnosis

**DOI:** 10.3389/fonc.2022.777066

**Published:** 2022-03-09

**Authors:** Dima El-Sharkawi, Ayoma Attygalle, Claire Dearden

**Affiliations:** ^1^ Department of Haematology, The Royal Marsden NHS Foundation Trust, London, United Kingdom; ^2^ Division of Molecular Pathology, The Institute of Cancer Research, London, United Kingdom; ^3^ Department of Histopathology, The Royal Marsden NHS Foundation Trust, London, United Kingdom

**Keywords:** diagnostics, mature T and NK-cell neoplasms, T-PLL, T-cell prolymphocytic leukemia, large granular lymphocyte (LGL) leukemia, adult T-cell leukemia/lymphoma (ATL), T cell lymphoma

## Abstract

T-cell clones can frequently be identified in peripheral blood. It can be difficult to appreciate whether these are benign and transient or whether they signify a clonal disorder. We review factors that aid in understanding the relevance of T-cell clones. Conversely, obvious pathological T-cell clones can be detected in blood, but there is uncertainty in how to categorize this clonal T cell population, thus, we adopt a multidisciplinary review of the clinical features, diagnostic material and radiology before making the diagnosis. In this review we shall discuss some of these challenges faced when diagnosing mature T-cell leukemias.

## Introduction

Mature T-cell neoplasms with leukemic involvement are rare and while many can present with archetypal features that allow for easy diagnostic categorization, other cases can be more difficult to sub-classify. Accurate and precise diagnosis requires integration of all the clinical findings along with morphological assessment, immunophenotyping, cytogenetic and molecular analysis of the peripheral blood, bone marrow and lymph node and radiology (1). A multi-disciplinary review of these cases is paramount to avoid incorrect diagnosis, for example, a histopathologist reviewing a lymph node biopsy may suggest a patient has nodal peripheral T-cell lymphoma in the absence of information regarding the white cell count and clinical picture (2).

In this review, we shall discuss some of the features that aid in subclassifying the mature T-cell leukemias and differentiating them from nodal peripheral T-cell lymphoma with leukemic involvement. We shall highlight rare examples of these diseases in order to avoid potential diagnostic pitfalls.

## What is the Differential for a Clonal T-Cell Population Identified in Peripheral Blood?

Lymphocytosis due to an increase in T-lymphocytes can easily be distinguished from clonal B-cell populations by basic flow cytometry methods. Once it has been established that the increase in lymphocytes are T cells, and further characterizing the T-lymphocyte population by morphology, immunophenotyping, cytogenetic and molecular analysis, an attempt to establish clonality is recommended, particularly in cases where the white cell count is low. This can be performed by several methods, namely, PCR-based methods, next generation sequencing (NGS) and flow cytometry of the TCR-V_β_ repertoire (albeit limited use in everyday practice) or more recently TRBC1 (3–7).

Persistent T-cell lymphocytosis and expansion of T-cell populations can be seen in many cases of chronic infection, for example HIV and CMV and indeed reactive to other malignancies (8–10). Similarly immune dysregulation due to primary immunodeficiencies or autoimmune conditions such as autoimmune lymphoproliferative disorders (ALPS) can lead to significant lymphoid proliferation and peripheral blood involvement. Thus, even in the absence of clonal T-cell expansion a persistent T-cell lymphocytosis may indicate significant pathology that requires multi-disciplinary team input both for both diagnosis and management. This discussion is beyond the scope of this paper. One important point to note is that many of these conditions in themselves increase the risk of lymphoma.

## Reactive Versus T-Cell Neoplasm?

The identification of a T-cell clone is not synonymous with a neoplasm. T-cell clones can be detected due to reactive causes, infection and senescence and can be persistent in these cases (11, 12). There is a spectrum of disorders both infective and autoimmune that can be associated with polyclonal expansion of T cells through to monoclonal expansion through to neoplastic proliferations of T cells. This is especially the case with large granular lymphocytic proliferations which can be seen in autoimmune conditions, and Felty’s syndrome, but similarly it is known that there is a strong link between Rheumatoid arthritis and LGLL. Furthermore, the pathogenesis of lymphoproliferative disorders, such as LGLL has been linked to chronic T cell activation with viruses such as HTLV or EBV implicated (13). This boundary and how we define these clones can be complex and can change with time (14). Furthermore, with improvements in diagnostics and also availability, there will be an increase in individuals identified with persistent T-cell clones with normal or even low lymphocyte counts. Interestingly, there is an association between clonal hematopoiesis (CHIP), MDS and clonal T-cell disorders. Not only do they share many of the same recurrent mutations seen predominantly in epigenetic regulators such as *DNMT3A* and *TET2*, suggestive that this may be early mutations in common progenitors, but they also often co-exist and there are numerous reports of co-existing MDS with LGLL or angioimmunoblastic T-cell lymphoma (15, 16).

T-cell clones of uncertain significance may be detected by molecular analysis solely, or there may be a small T-cell population identified by flow cytometry often with a large granular lymphocyte phenotype (as described below) (17, 18). While there is no equivalent to monoclonal B-lymphocytosis, many of these incidental clones can be considered as ‘T-cell clones of uncertain significance’ if the criteria for diagnosis of large granular lymphocytic leukemia (LGLL) or other mature T-cell leukemia are not met. However, the significance of the T-cell clones in the context of cytopenias and therefore how to manage them is not clear (19). It should also be noted that large granular lymphocytic proliferations can be seen with other hematological and non-hematological conditions, namely, myelodysplasia, plasma cell dyscrasias, aplastic anaemia, post- stem cell transplant, HIV infection and treatment with dasatinib (20–24). The presence of mutations in *STAT3* and *STAT5b* does not immediately define a diagnosis of LGLL as these mutations are not specific to this disease (14). Thus, if patients do not have sufficient evidence for a positive diagnosis of a defined T-cell leukemia, then we prefer to consider them as having T-cell clones of uncertain significance. Akin to MGUS and monoclonal B cell lymphocytosis of uncertain significance, these should however be followed up as these clones may acquire secondary events that drive progression and develop into malignancy (25). Our preference depending on clinical situation is to monitor patients every 6 months in the first instance and then annually if no progression occurs.

## Acute Versus Mature T-Cell Neoplasm?

While distinguishing between T-lymphoblastic leukemia (T-ALL) and mature T-cell neoplasms is usually very straightforward with the presence of immature markers such as CD1a, CD34, and TdT in the former, there have been unusual cases of T-prolymphocytic leukemia, T-PLL seen with lack of surface CD3 and CD45 that can make the diagnosis more difficult (26, 27). Similarly, there have been reports of mature T-cell neoplasms aberrantly expressing immature markers, such as CD1a (28, 29).

## Mature T Cell Leukemia With Nodal/Cutaneous Involvement Versus Nodal/Cutaneous T Cell Lymphoma With Leukemic Involvement?

The mature T-cell leukemias are sufficiently diverse from one another that they are usually readily discernible; however distinguishing them from nodal or cutaneous lymphomas with leukemic involvement can be challenging and thus requires integration of all results before reaching a diagnosis, occasionally this can require multiple biopsies and can take time before a conclusion can be made (**Table 1**).

**Table 1:** Summary of the defining features of the mature T cell leukemias.

	T-PLL	T-LGLL	ATLL	SS
Classic Clinical Features	**Rapidly progressive** High white cell count Lymphadenopathy, splenomegaly, skin involvement **effusions**	**Indolent** Often clone is modest size <20 × 10^9^/L Associated cytopenias Associated autoimmune history	**Presence of HTLV1** Variable involvement by skin, nodes, blood, marrow and extranodal disease **Hypercalcemia**	**Erythroderma** Generalized lymphadenopathy Pruritus
Morphology	**Basophilic prolymphocytes with cytoplasmic blebbing** Small cell (20%) and Sezary (5%) variants	**Large granular lymphocytes**	Variable **“Flower cells”**	**Cerebriform cells**
Typical Immunophenotyping (rare variations do exist for all diagnoses)	CD2^+^, CD3^+^, CD5^+^, **CD7^++^** CD4/8 variable CD1a^−^, TdT^−^, CD25^−/+^ **TCL1^+^**	CD2^+^ CD3^+^ CD5^+^ **CD8^+^** **CD56^+^ CD57^+^** (NK and CD4^+^ and δγ cases also seen)	CD2^+^ CD3^+^ CD5^+^ **CD4^+^ CD25^+^** **CD7−**	CD2^+^ CD3^+^ CD5^+^ CD4^+^ **CD7− CD26−**
Specific molecular or cytogenetic aberration	**t(14,14); inversion 14; t(X,14);** iso8q; complex cytogenetics	***STAT3* and *STAT5b mutations***	High frequency of mutations	Non-specific and heterogeneous pattern of translocations and mutations

Those listed in bold can be helpful in differentiating from each other as are quite specific to that disease category.

## Large Granular Lymphocytic Leukemia

Large granular lymphocytic leukemia (LGLL) typically presents with cytopenias, most commonly neutropenia. Median age at presentation is 66 years (30–32). There is a strong association with autoimmune conditions, and approximately 15% of patients with LGLL will also have rheumatoid arthritis.

### Morphology

Typically there are no dysplastic features on the peripheral blood unless there are co-existing conditions and the cytopenias are evident. The large granular lymphocytes tend to be infrequent but are characteristic large lymphocytes with abundant cytoplasm and azurophilic granules. The distribution of the lymphocytes is intrasinusoidal in the bone marrow trephine that is otherwise normo- or hypercellular.

### Immunophenotyping and Molecular Analysis

The characteristic immunophenotypic profile of LGLL is that of mature cytotoxic T cells most commonly αβTCR, CD2, CD3, CD8, CD56, and CD57 positive. Less commonly, LGLL can be comprised of CD4 positive T-cells, NK cells (classified as chronic lymphoproliferative disorder of NK cells in the most recent WHO classification), or have a γδTCR (33). While these can all be readily differentiated from T-LGLL by flow cytometry, it is important to consider their differential diagnoses such as aggressive NK cell leukemia or hepatosplenic T cell lymphoma, especially if patents have a more aggressive clinical picture.

Clonality may be assessed by flow cytometry using TRBC1 or more commonly by assessing for TCR gene rearrangements. Molecular analysis of *STAT3* and *STAT5b* can be helpful as recurrent mutations in these genes have been identified in LGLL, but are not specific.

### Making the Diagnosis

The WHO diagnostic criteria for LGLL are defined as a persistent (>6 months) increase in the number of peripheral blood large granular lymphocytes, usually 2–20 × 10^9^/L without a clearly defined cause (33). However, it is stated that LGL counts of less than 2 × 10^9^/L that otherwise meet the diagnosis are still consistent.

Hence, this diagnosis can only be made once persistence of the clone has been demonstrated. Often the authors are asked to review cases where patients have been investigated for cytopenias and while there are persistent T-cell rearrangements identified by molecular analysis, there is no associated lymphocyte population with LGLL phenotype identified by flow cytometry or infiltrate seen on bone marrow trephine. In these cases we would suggest continued infrequent monitoring and reassessment if the clinical situation changes but that this does not meet the diagnostic criteria for LGLL (34).

We have seen cases with very high white count with lymphocytes >100 × 10^9^/L and so while low level clones are more common, they are not a defining feature.

Similarly, rare patients have presented with a predominantly nodal distribution of disease and this must not be assumed to be PTCL NOS based on distribution alone.

Cases of LGLL with more unusual immunophenotypic profiles such as γδTCR can lead to other differential diagnoses such as gamma delta hepatosplenic T-cell lymphoma (35, 36). However, by combining the clinical features such as generalized symptoms, rapidity of onset of symptoms, presence of hepatosplenomegaly, and bone marrow sinusoidal expansion by lymphoma cells the two can be readily distinguished, emphasizing that the pathologist cannot make the diagnosis in isolation, without knowing the clinical picture.

## T-Prolymphocytic Leukemia

T-prolymphocytic leukemia (T-PLL) characteristically presents at a median age of 65 years. Patients with ataxia telangiectasia have an increased risk of T-PLL and in these cases, the presentation can be in their 20s. Often the illness presents rapidly, with a rapidly rising white cell count, with generalized symptoms and also effusions, ascites, edema, and peri-orbital edema and skin infiltration. However, T-PLL can have an indolent pre-phase that is detected incidentally, when patients will not have these symptoms and have smaller more stable clones but with the characteristic phenotype as described below.

### Morphology

The morphology can be variable with three characteristic appearances described. These include the more typical prolymphocytes with blebbing of the cytoplasm and single nucleolus; small cell variant with cells displaying condensed chromatin and nucleoli invisible by light microscopy; and cerebriform variant with an irregular nuclear outline similar to the lymphocytes seen in Sézary syndrome.

### Immunophenotyping

The lymphocytes are post-thymic and express mature markers positive for CD2, CD3, CD5 and CD7 and CD52. CD4^+^ CD8^−^ is most commonly seen, with rarer cases expressing only CD8 or double positive. The latter is quite specific to T-PLL compared to other mature T cell leukemias, and so can be helpful for making the diagnosis. Similarly, TCL1 expression can be assessed by flow cytometry and is specific to T-PLL.

### Cytogenetics and Molecular Analysis

Changes involving chromosome 14 are the most common genetic alteration, seen in over 90% of cases. Inv(14)(q11q32) and t(14;14)(q11;q32) causes juxtaposition of TCRα and TCL1 or TCL1B leading to activation (37). This rearrangement can be identified by FISH (karyotype has a lower sensitivity), and the aberrant TCL1 protein expression can also be detected by flow cytometry or immunohistochemistry (38). The translocation t(X;14) is present in approximately 10–20% cases and involves the rearrangement of the TCRα locus with the proto-oncogene MTCP1 (39–41).

Other cytogenetic abnormalities are also commonly found, namely, abnormalities of chromosome 8 which often results in increased expression of MYC, deletions in 11q23, 12p, 22q, and 17 or abnormalities in chromosome 6 have also been identified with the majority of patients exhibiting a complex karyotype (37, 40, 42–47). While molecular analysis is also performed, the recurrent mutations in genes such as *ATM*, *JAK3*, and *STAT5b* are not specific (40).

### Making the Diagnosis

As well as assessing for TCL1 expression, in our center we will also perform FISH to look for the characteristic inversion 14(q11q32) or t(14;14)(q11q32), but importantly also perform cytogenetics to look for other aberrations that are frequently seen in T-PLL.

International consensus criteria have been recently published to guide the diagnosis (48). When specific cytogenetic aberrations or protein expression are detected, the diagnosis is certain; however, there is a small subset of cases which have been diagnosed elsewhere to have T-PLL on the basis of a leukemic clonal T-cell population “compatible” with T-PLL by flow cytometry and with involvement by a “T-PLL specific site” that on further investigation with a wider T-cell panel, has been reclassified as PTCL-NOS due to lack of cytogenetic aberrations, and features that would be more unusual for T-PLL such as weak CD7.

Identification of CD4^+^ CD8^+^ double positive T-cell populations, can be very suggestive of the diagnosis of T-PLL. While ATLL is characteristically CD4^+^ CD25^+^, CD25 expression can also be seen in T-PLL and so does not differentiate between the two, HTLV analysis aids in differentiation of these cases. Typically Sézary cells do not express CD7, which can help diagnostically. A recent case that had been referred to our center as possible T-PLL was in a patient with marked erythroderma and a relatively modest lymphocytosis, in this case the weak CD7 positivity pushed the referring center to this diagnosis, however, the clinical history of the erythroderma being significant for many years and the discrepancy of the extent of cutaneous involvement and progression with lack of progression of the leukemic component made the diagnosis of T-PLL very unlikely. Skin biopsy showed evidence of a CD4 positive T-cell lymphoma infiltrate with small to intermediate sized T-cell infiltrate with focal epidermotropism. This in conjunction with the lack of any specific cytogenetic aberration by FISH analysis for TCRAD break-apart allowed the regional skin lymphoma unit and our center to conclude that this was most in keeping with Sézary syndrome. This highlights the importance of multidisciplinary involvement in difficult cases such as these.

Similarly, cases can be seen where nodal lymphomas are incorrectly diagnosed as T-PLL due to the leukemic involvement or the converse when initially the patient presents with lymphadenopathy but the lymphocytosis is not such a feature or perhaps in a more indolent phase (49, 50).

## Adult T-Cell Leukemia/Lymphoma (ATLL)

Despite the marked heterogeneity of this disease, given the knowledge of the etiological infectious agent, HTLV1, differentiating this from other mature T cell neoplasms is generally easier than other mature T cell leukemias. However, HTLV1 serology should be undertaken in any case of cutaneous, nodal or leukemic mature T-cell neoplasm in order not to miss this diagnosis (51).

### Morphology

Several morphological variants have been described with the archetypal variant being medium to large sized “flower cells” with nuclear indentations (33, 52).

### Immunophenotype

Mature T-cell markers are expressed, namely, CD2, CD3, and CD5, but usually lack CD7. The majority are CD4 positive and CD8 negative but CD8 positive and double positive cases have been described (33, 53). CD25 is strongly expressed in nearly all cases. CD30 expression is variable.

### Cytogenetics and Molecular Analysis

The genomic landscape of ATLL is complex with a high frequency of mutations with regional variations and variations dependent on subtype of ATLL (54–56). Most frequently mutated genes are *PLCG1*, *PRKCB*, *VAV1*, *IRF4*, *FYN*, *CARD11*, and *STAT3*.

### Sézary Syndrome

Sézary syndrome usually presents in patients in the older (60 years plus) age group. Symptoms most frequently include erythroderma and generalized lymphadenopathy. The classical triad of erythrodermic pruritic rash covering >80% of the body surface area, lymphadenopathy and circulating Sézary cells can aid in diagnosis (33, 57). The history is usually quite short, due to the rapid progression, however a secondary Sézary syndrome occurring following a more prolonged history with documented preceding mycosis fungoides has also been defined (57).

### Morphology

Sézary cells in the peripheral blood typically show cerebreiform nuclei. Skin changes histologically are similar to mycosis fungoides with less epidermotropism. Effacement of the lymph nodes with dense monotonous infiltrates can be seen (33).

### Immunophenotype

The immunophenotype of the cells usually are CD3 and CD4 positive and lack CD7 and CD26. Rarer phenotypes have been seen such as loss of other T cell antigens, CD4 negative CD8 positive disease or double positive (57)

### Cytogenetic and Molecular Analysis

The cytogenetic rearrangement seen are non-specific with complex cytogenetics and numerous mutations identified in studies exploring the molecular landscape (58, 59).

### Making the Diagnosis

It is important to analyze all compartments (skin, peripheral blood, lymph nodes) for possible involvement. Histological changes in the skin can be non-specific and thus numerous skin biopsies are frequently taken before a diagnosis is made. Furthermore, many inflammatory skin conditions may lead to reactive T cell clones in the peripheral blood which can complicate diagnosing the skin condition. This emphasizes the importance of peripheral blood analysis which can help confirm the diagnosis. Skin biopsy and peripheral blood show the same TR gene rearrangements. The total Sezary count is greater than 1 ×10^°^/L with an expanded CD4 positive population resulting in CD4:CD8 ratio of greater than 10 with loss of CD7 being quite characteristic (2, 33).

### Nodal Lymphoma With Leukemic Involvement

All nodal lymphomas have been reported to have leukemic involvement in rare cases (60–62). The diagnosis of these would not be performed on peripheral blood alone and would need to be correlated with the bone marrow, lymph node histology and any clinical information. We have had a number of cases referred for second opinion on diagnosis with quite marked T-lymphocytosis, who are ultimately classified as PTCL NOS due to lack of defining markers to suggest an alternative diagnosis.

## Conclusions

Not all patients who present with mature T-cell leukamias have easily classifiable disease, and in these cases, if they do not fulfil the currently recognized diagnostic categories, by definition they need to be considered as peripheral T cell lymphoma, not otherwise specified. As our use of next generation sequencing, and gene expression and methylation profiling increases, how we define these neoplasms is likely to change and improve. In the meantime, the integration of clinical, morphological, genetic and histopathological features is paramount to ensure that optimal management is employed to avoid under- or over-treatment of the patient.

## Author Contributions

DE, AA, and CD devised concept of article. DE wrote the first draft. All authors listed have made a substantial, direct, and intellectual contribution to the work and approved it for publication.

## Conflict of Interest

The authors declare that the research was conducted in the absence of any commercial or financial relationships that could be construed as a potential conflict of interest.

## Publisher’s Note

All claims expressed in this article are solely those of the authors and do not necessarily represent those of their affiliated organizations, or those of the publisher, the editors and the reviewers. Any product that may be evaluated in this article, or claim that may be made by its manufacturer, is not guaranteed or endorsed by the publisher.
